# Pan-Cancer Analysis Reveals the Relation between TRMT112 and Tumor Microenvironment

**DOI:** 10.1155/2022/1445932

**Published:** 2022-08-30

**Authors:** Haitao Xu, Caihong Jiang, Fusheng Yao, Hong Liang, Hong Yan, Dangui Chen, Youzhi Wu, Long Zhong

**Affiliations:** ^1^Department of Hematology, Anqing Municipal Hospital, Anqing Medical Center Affiliated to Anhui Medical University, Anqing, China; ^2^Department of Pediatric Surgery, Anqing Municipal Hospital, Anqing Medical Center Affiliated to Anhui Medical University, Anqing, China

## Abstract

Dysregulated epigenetic modifications play a critical role in cancer development where TRMT112 is a member of the transfer RNA (tRNA) methyltransferase family. Till now, no studies have revealed the linkage between TRMT112 expression and diverse types of tumors. Based on TCGA data, we first probed into the relation between TRMT112 and prognosis and the potential role of TRMT112 in tumor microenvironment across 33 types of tumor. TRMT112 presented with increased expression in most cancers, which was significantly prognostic. Furthermore, TRMT112 was associated with tumor-associated fibroblasts in a variety of cancers. Additionally, a positive relationship was identified between TRMT112 expression and multiple tumor-related immune infiltrations, such as dendritic cells, CD8+ T cells, macrophages, CD4+ T cells, neutrophils, and B cells in lung adenocarcinoma and breast invasive carcinoma. In summary, our results suggest that TRMT112 might be a potential prognostic predictor of cancers and involved in regulating multiple cancer-related immune responses to some extent.

## 1. Introduction

Epigenetic modifications have been demonstrated to play an important role in cancer development, whose dysregulation can alter the expression of tumor suppressors and activators [1]. For example, histone modifications are the most common modifications, which can regulate transcription activation and repression [2]. Histone methylation has a key role in cell development and differentiation under a dynamic process. Aberrant histone methylations are found to be related to tumorigenesis [2]. With the exploration of the critical role of aberrant methylations in cancer development, methyltransferase inhibitors are considered pharmacological drugs for cancer treatment through activating tumor suppressor genes [3].

Transfer RNA (tRNA) methyltransferase subunit 11-2 (TRMT112), a small evolutionarily conserved protein, is a cofactor of diverse methyltransferases implicated in ribosomal RNA (rRNA), DNA, tRNA, and protein methylation [4–6]. THUMPD3 extensively catalyzes the modification of N2 methylguanosine (m2G) at positions 6 and 7 of transfer RNA (tRNA) with the help of methyltransferase activating helper protein (TRMT112) [7]. It shows relationships to a minimum of 4 methyltransferases (such as N6AMT1 and WBSCR22) [8, 9] and is necessary for the maintenance of enzyme activity and stability in mammalian cells. Besides, TRMT112 is evidently associated with the proliferation of androgen receptor-dependent cells, especially enzalutamide-resistant prostate cancer cells and xenograft tumors [10]. Unfortunately, none of the studies comprehensively revealed the linkage between TRMT112 expression and pan-cancer. Therefore, we attempted to characterize the relationship between TRMT112 and cancer development to explore its potential as a therapeutic target in cancer.

Given that tumorigenesis is a complex process, pan-cancer analysis appears to be critical in analyzing any target gene. In the meantime, the associations with clinical outcomes and the underlying mechanisms are also being placed in a significant position. The Cancer Genome Atlas (TCGA), funded by the government, contains functional genomic data for a variety of tumors [11], which enables us to perform pan-cancer analysis. With the help of bioinformatics analysis, a number of gene signatures have been developed for predicting cancer prognosis and assisting cancer treatment [12–14].

Here, we initially explored the pan-cancer patterns of TRMT112 expression by using the TCGA project and GTEx database [15]. Furthermore, the underlying molecular mechanisms linking TRMT112 expression with the initiation or clinical prognosis of diverse tumors were investigated by analyzing several factors, including gene expression, immune infiltration, survival outcome, and related cellular pathways.

## 2. Bioinformatics Analysis

### 2.1. Gene Expression Pattern

The TRMT112 mRNA expression pattern in diverse tumors was examined utilizing the Oncomine database (https://www.oncomine.org) with *P* < 0.001 and fold change >1.5. Besides, RNAseq data in TPM format from tumor and matching normal samples, respectively, documented in TCGA (https://portal.gdc.cancer.gov/) and GTEx databases, which were unified and processed by the Toil process [16], were collected from UCSC Xena (https://xenabrowser.net/datapages/) and then transformed by base-2 logarithms (log2). Subsequently, TRMT112 expression in tumor and normal samples was examined and compared in *R* (version 3.6.3) software (Student's *t*-test). Moreover, TRMT112 expression data corresponding to various pathologic stages (stages I, II, III, and IV) were extracted from the GEPIA2 (v2) pane “Pathological Stage Plot” (http://gepia2.cancer-pku.cn/#analysis) [17]. The TRMT112 expression data were conversed by log2 (TPM+1) and were applied to box plots.

UALCAN (http://ualcan.path.uab.edu/index.html) is a platform available for cancer data analysis, and it provides protein expression analysis for the dataset from the National Cancer Institute's Clinical Proteomic Tumor Analysis Consortium (CPTAC) [18]. In this study, we used UALCAN to obtain available datasets for six types of tumors, namely, colon cancer, clear cell renal carcinoma, ovarian cancer, breast cancer, lung adenocarcinoma (LUAD), and uterine corpus endometrial cancer (UCEC). Additionally, the difference in TRMT112 proteins between primary tumors and normal tissue was investigated. The bioinformatics analysis of this study was supported by SangerBox (http://vip.sangerbox.com/) [19].

### 2.2. Survival Outcome

The survival data of TRMT112 for disease-free survival (DFS) and overall survival (OS) in all TCGA tumors were extracted from the “Survival Plots” pane of GEPIA2. Cancer patients were assigned to the high and low-expression cohorts according to the median TRMT112 expression level. The hypothesis was tested utilizing a log-rank test. The “Survival Analysis” function of GEPIA2 was utilized to construct the survival plots. Besides, further COX regression models were established to determine the link between clinical characteristics and survival outcomes in head and neck squamous cell carcinoma (HNSC) RNAseq data in the TCGA database.

### 2.3. Immune Infiltration

The “Gene Module” under “Immune Association” of the TIMER (https://cistrome.shinyapps.io/timer/) website was visited to extract the link of TRMT112 expression with tumor-related fibroblasts in all TCGA tumors, followed by the Spearman rank correlation test with calculations of partial correlation (cor) values and *P* values. Scatter plots and heat maps were utilized to provide a visual representation of the data. Additionally, the link of TRMT112 expression with the infiltration status of 6 immune cells, namely, dendritic cells, CD4+ T cells, macrophages, CD8+ T cells, neutrophils, and B cells, was examined. In the meantime, the association with tumor purity was identified as well.

### 2.4. TRMT112-Related Gene Set Enrichment

The STRING web platform (https://string-db.org/) was visited for protein-protein interaction (PPI) analysis. The following criteria were used to search for TRMT112's target-binding proteins: active interaction sources (“Experimentes”), the maximum number of interactors to show (“no more than 50 interactors” in the 1st shell), the meaning of network edges (“Evidence”), and the minimum required interaction score (“Low Confidence (0.150)”).

The “Similar Genes Detection” function of GEPIA2 was applied to acquire the topmost 100 TRMT112-related target genes across all datasets of TCGA tumors and normal samples, and the “Correlation Analysis” function was utilized to characterize the correlation of TRMT112 expression with selected genes using the Pearson analysis. Additionally, heat map data for the chosen genes were acquired using the TIMER2 (http://timer.cistrome.org/)module “Gene_Corr.” The corr values and *P* values were derived with the help of the Spearman rank test.

To obtain the genes that both bind and interact with TRMT112, the VennDiagram *R* package (https://cran.r-project.org/web/packages/VennDiagram/index.html) was used to construct a Venn diagram for cross-analysis.

The DAVID (https://david.ncifcrf.gov/) tool was utilized for analyses of Gene Ontology (GO) annotation and the Kyoto Encyclopedia of Genes and Genome (KEGG) pathways, followed by the ggplot2 package applied for visualization.

## 3. Results

### 3.1. Analysis of Gene Expression

**Figure 1:**
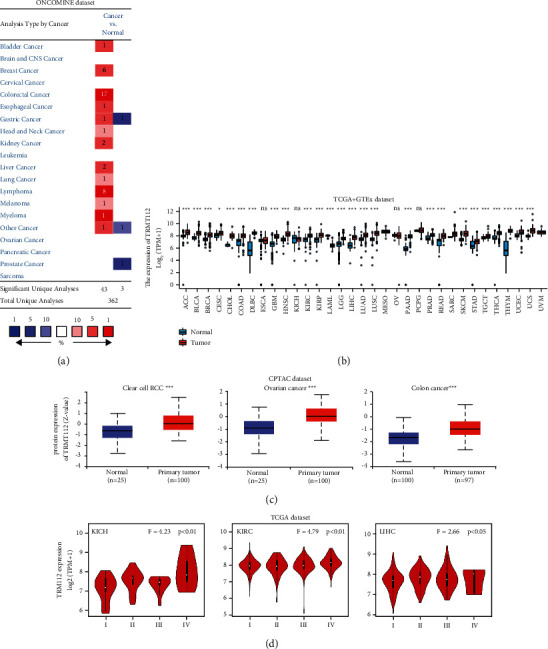
TRMT112 gene expression in distinct cancers and pathologic stages. (a) TRMT112 expression in several kinds of malignancies and in normal samples gathered from the Oncomine database. Red and blue indicate high and low TRMT112 expression in cancer, respectively. (b) TRMT112 expression in tumor specimens gathered from the TCGA database and in matching normal specimens from the GTEx database. No expression data of normal samples were provided in MESO and UVM. Student's *t*-test was performed. ns, *P* ≥ 0.05; ^*∗*^*P* < 0.05; ^*∗∗*^*P* < 0.01; ^*∗∗∗*^*P* < 0.001. (c) TRMT112 protein expression in normal and malignant samples of colon cancer, clear cell renal carcinoma, and ovarian cancer premised on the CPTAC dataset. Student's *t*-test was performed. ^*∗∗∗*^*P* < 0.001. (d) The TRMT112 expression in KICH, KIRC, and LIHC premised on the pathologic stage (stages I, II, III, and IV). Log2 (TPM+1) is applied to the logarithmic value. ANOVA was performed.

We first visited the Oncomine platform to explore the TRMT112 mRNA expression pattern in pan-cancer. As compared to the respective normal control, TRMT112 presented with profoundly elevated expression in multiple cancers, particularly bladder cancer, melanoma, breast cancer, lymphoma, colorectal cancer, lung cancer, esophageal cancer, stomach cancer, myeloma, liver cancer, kidney cancer, and head and neck cancer (Figure 1).

To additionally identify the TRMT112 expression in pan-cancer, corresponding RNAseq data were extracted from TCGA and GTEx databases. As illustrated in Figure 1(b), TRMT112 displayed a decreased expression in tumor tissues of acute myeloid leukemia (LAML). On the contrary, an increased expression was noted in most cancers except for uveal melanoma (UVM), ovarian serous cystadenocarcinoma (OV), pheochromocytoma and paraganglioma (PCPG), sarcoma (SARC), kidney chromophobe (KICH), and esophageal carcinoma (ESCA) (Figure 1(b)).

Results of the CPTAC data showed that TRMT112 proteins in colon cancer, ovarian cancer, and clear cell renal cell carcinoma samples were more abundant relative to that in the respective normal control (Figure 1(c), *P* < 0.001).

Afterward, the HEPIA2 function “Pathological Stage Plot” was applied to characterize the correlation of TRMT112 expression with cancer pathologic stages, and significant associations were noted in KICH, liver hepatocellular carcinoma (LIHC), and kidney renal clear cell carcinoma (KIRC) (Figure 1(d)).

### 3.2. Survival Data

**Figure 2:**
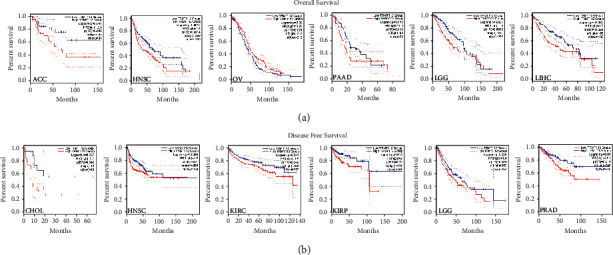
Relationship of TRMT112 gene expression with the survival of malignancies from the TCGA database. GEPIA2 was utilized to analyze overall survival (OS) (a) and disease-free survival (DFS) and (b) premised on TRMT112 expression in various malignancies from the TCGA database. The Kaplan–Meier curve and survival graph are shown.

To assess the survival significance of TRMT112, the patients were assigned to comprise two groups according to TRMT112 expression from TCGA data. Higher TRMT112 expression predicted poorer OS among patients with adrenocortical carcinoma (ACC, *P*=0.046), HNSC (*P*=0.0014), lower-grade brain glioma (LGG, *P*=0.0012), LIHC (*P*=0.0028), and pancreatic adenocarcinoma (PAAD, *P*=0.018) (Figure 2). Additionally, lower TRMT112 expression predicted shorter OS time in patients with OV (*P*=0.021).

As regards DFS, similar results were obtained, which demonstrated a relationship between the higher TRMT112 levels and the poorer DFS in cholangiocarcinoma (CHOL, *P*=0.042), KIRC (*P*=0.042), kidney renal papillary cell carcinoma (KIRP, *P*=0.02), HNSC (*P*=0.01), LGG (*P*=0.029), and prostate adenocarcinoma (PRAD, *P*=0.011) (Figure 2(b)).

### 3.3. The Relation between TRMT112 Expression and HNSC Prognosis

**Figure 3:**
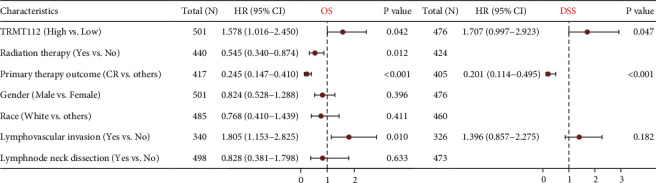
Forest plot of multivariate COX regression analysis for disease-specific survival (DSS) and overall survival (OS) in head and neck squamous cell carcinoma (HNSC).

In the previous section, we observed that high and low TRMT112 groups had significant differences in both overall survival and disease-free survival only in HNSC. From the aspect of prognostic significance in HNSC, the survival *R* package was utilized to detect the link between TRMT112 expression, clinical characteristics, and survival in TCGA-HNSC data. In the univariate COX analysis, TRMT112 expression, radiotherapy, initial treatment effect, and lymphatic vascular infiltration were related to OS; and TRMT112 expression and initial treatment effect were related to disease-specific survival (DSS) (Tables S1 and S2). In the further multivariate analysis, high TRMT112 expression was proven to be independently prognostic for worse OS and DSS (HR = 1.578, 95% CI = 1.016–2.450, *P*=0.042; HR = 1.707, 95% CI = 0.997–2.923, *P*=0.047) (Tables S1 and S2 and Figure 3).

### 3.4. Immune Infiltration Analysis

**Figure 4:**
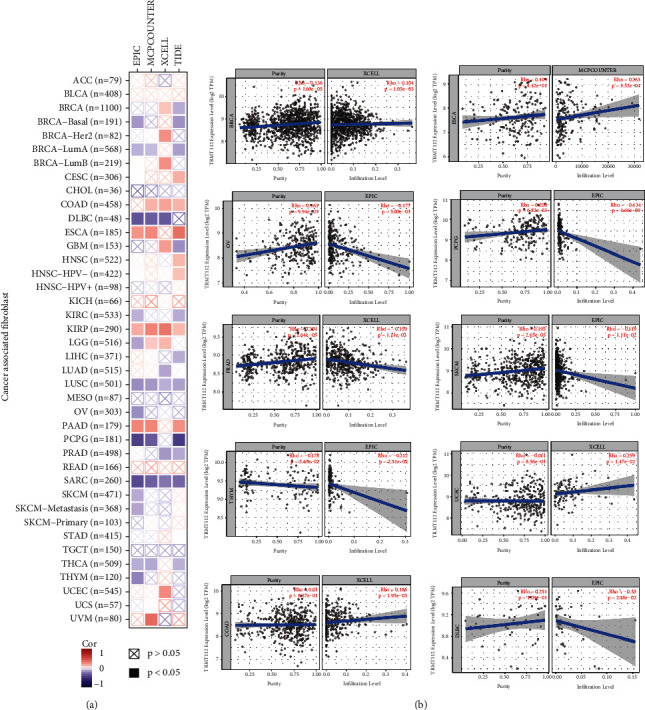
Relationship between TRMT112 expression and tumor-related fibroblasts. The possible link between TRMT112 expression and the tumor-associated fibroblasts in distinct TCGA cancer types through various algorithms.

Being important in the tumor microenvironment (TME), immune infiltrates are significantly involved in the initiation, development, or metastasis of cancer [20, 21]. In addition, tumor-associated fibroblasts in the TME have been proven to participate in regulating the function of diverse immune infiltrates in tumors [22, 23]. Here, the relationship between the immune infiltrates of fibroblasts in distinct TCGA tumors and the TRMT112 expression was investigated through several algorithms, including EPIC, CIBERSORT-ABS, MCPCOUNTER, XCELL, QUANTISEQ, CIBERSORT, and TIMER. A positive association was demonstrated as regards the TRMT112 expression and the tumor-related fibroblasts in BRCA (*R* = 0.104), ESCA (*R* = 0.263), UCEC (*R* = 0.259), and COAD (*R* = 0.186); while TRMT112 level was negatively linked to tumor-related fibroblasts in OV (*R* = −0.177), PCPG (*R* = −0.434), skin cutaneous melanoma (SKCM) (*R* = −0.119), PRAD (*R* = −0.159), thymoma (THYM) (*R* = −0.212), and lymphoid neoplasm diffuse large B cell lymphoma (DLBC) (*R* = −0.350) (Figure 4). The above results suggested that TRMT112 had a correlation with fibroblast infiltration, but the correlation strength varied in different cancer types.

### 3.5. Correlation between TRMT112 Expression and Immune Infiltrates in LUAD and BRCA

**Figure 5:**
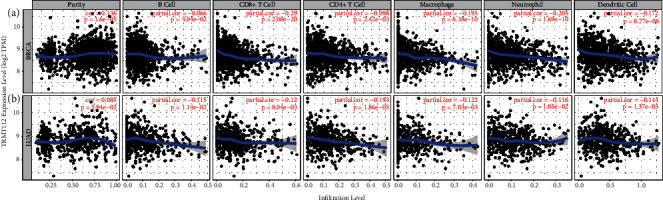
Relationship between TRMT112 expression and the infiltration status of immune cells in BRCA and LUAD. (a) The expression of TRMT112 shown to have a substantial positive link to tumor purity and infiltration levels of dendritic cells, CD4+ T cells, neutrophils, macrophages, CD8+ T cells, and B cells in BRCA. (b) The TRMT112 expression level independent of tumor purity in LUAD but positively linked to various tumor-infiltrating immune cells. *P* < 0.05, judged to have statistical significance.

Six typical immune cells were selected to evaluate the link between TRMT112 and the infiltration status of immune cells in LUAD and BRCA by the TIMER tool. Positive associations were indicated between the TRMT112 expression and the infiltration status of dendritic cells, neutrophils, CD4+ T cells, macrophages, CD8+ T cells, and B cells in LUAD and BRCA (Figure 5). Moreover, the TRMT112 expression level was independent of tumor purity (*R* = 0.085, *P*=5.94*E* − 2) in LUAD, but presented with a positive link to tumor purity (*R* = 0.136, *P*=1.6*E* − 05) in BRCA. These results imply that TRMT112 might influence the survival of patients with LUAD and BRCA by interacting with tumor-infiltrating immune cells.

### 3.6. Analysis on TRMT112-Related Genes

**Figure 6:**
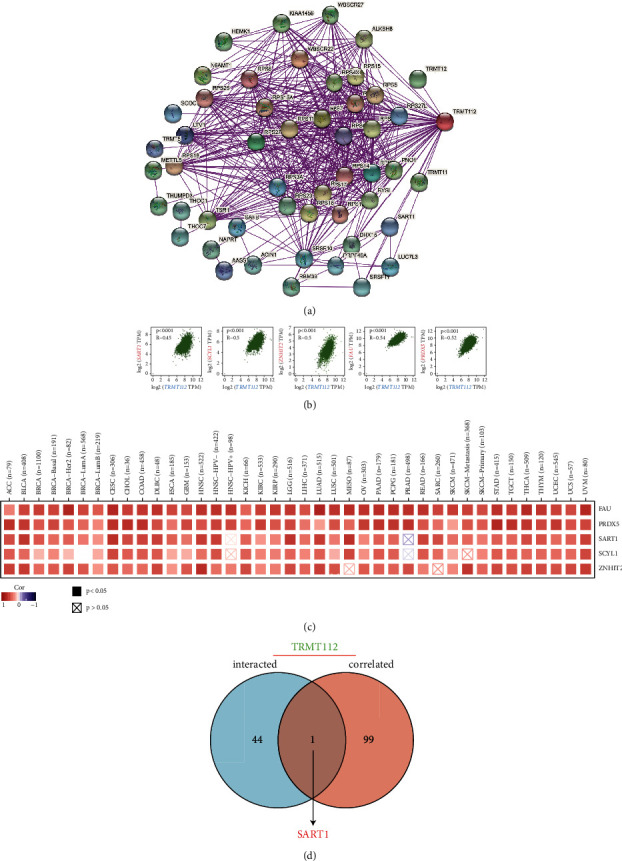
TRMT112-related protein and gene analysis. (a) STRING utilized to retrieve all of the TRMT112-binding proteins that have been experimentally determined before. (b) The topmost 100 TRMT112 expression-associated genes obtained in all cancers in the TCGA data through the GEPIA2 program. We examined the link between the expression of TRMT112 and the expression of a series of target genes selected (SART1, SCYL1, ZNHIT2, FAU, and PRDX5). (c) Corresponding heat map data based on diverse types of cancers. (d) The intersection analysis of TRMT112-binding genes and TRMT112-related genes.

**Figure 7:**
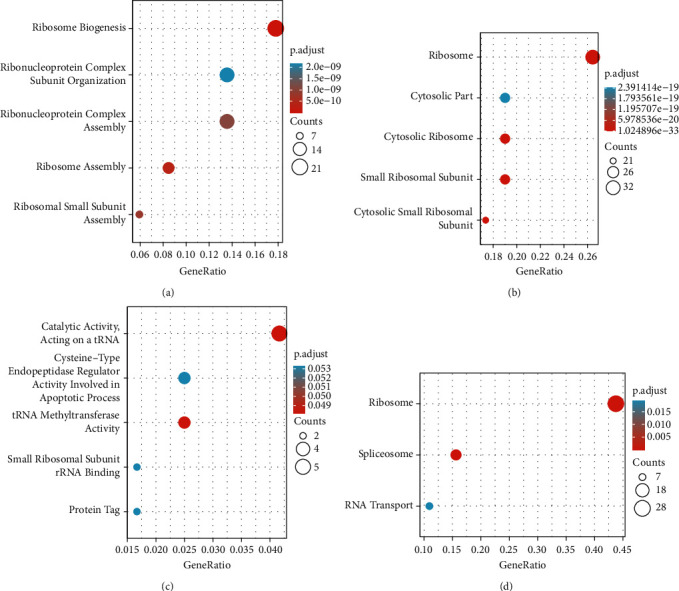
GO annotation and KEGG enrichment analyses of TRMT112-associated genes. Analysis of GO annotation and KEGG pathway enrichment premised on TRMT112-binding genes and TRMT112 expression-related genes. (a) Biological process. (b) Cellular components. (c) Molecular function. (d) KEGG pathway.

To clarify the molecular mechanism by which TRMT112 participates in tumorigenesis, TRMT112-binding protein, and TRMT112 expression-related genes were explored. We identified 50 available binding proteins through the use of the STRING tool.  Figure 6 shows the PPI network. Furthermore, the top 100 TRMT112 expression-associated genes were obtained from the TCGA data through GEPIA2. The findings illustrated that TRMT112 expression was linked to SART1 (*R* = 0.45), SCYL1 (*R* = 0.5), ZNHIT2 (*R* = 0.5), FAU (*R* = 0.54), and PRDX5 (*R* = 0.52) in a positive manner (Figure 6(b)). The results were also demonstrated in a majority of cancer types based on the matching heat map data (Figure 6(c)). In addition, the intersection analysis data showed that there was a common member, namely, SART1 in the above two groups (Figure 6(d)). Furthermore, we performed GO annotation and KEGG enrichment analyses by combining the two above datasets. In the GO analysis, most of the TRMT112-related genes were related to the RNA metabolism pathway or cell biology, such as the catalytic activity of RNA, cytosolic small ribosomal subunit, ribosome synthesis, ribosomal small subunit assembly, small ribosomal subunit rRNA binding, and ribosome assembly. Moreover, KEGG analysis illustrated that TRMT112 could perform an integral function in tumor pathogenesis with the involvement of ribosome and RNA transport (Figure 7).

## 4. Discussion

Human TRMT112 protein, a homolog of *Saccharomyces cerevisiae* TRM112 (tRNA methyltransferase 11-2), serves as a cofactor for diverse methyltransferases implicated in rRNA, tRNA, and protein methylation [24, 25]. As an evolutionarily conserved protein, TRMT112 interacts with WBSCR22 and ALKBH8 proteins and shows relationships with multiple cell biological processes, including cell proliferation and DNA damage [26, 27]. Earlier research reports noted that TRMT112 affected the proliferative activity of enzalutamide-resistant prostate cancer cells and the growth of xenograft tumors [10]. Nonetheless, it is yet unclear if TRMT112 is involved in the onset of different types of cancers via certain common molecular processes. Recognizing that there has been no previous study examining TRMT112 over a broad range of tumor types, we conducted a thorough analysis to evaluate TRMT112 across 33 tumor types utilizing data from the TCGA, CPTAC, and GTEx as well as data on gene expression, survival, and immune infiltration.

We observed that TRMT112 showed upregulated expression in many types of tumors. High TRMT112 expression was linked to the adverse OS in HNSC, PAAD, ACC, LGG, and LIHC. Additionally, high TRMT112 expression was also a risk indicator for unfavorable DFS in CHOL, HNSC, KIRC, KIRP, LGG, and PRAD. Notably, the expression of TRMT112 was found to correlate with both OS and DFS in HNSC. In this context, the relationship between TRMT112 and survival of HNSC patients was further investigated with clinical characteristics included in univariate and multivariate COX analyses, which indicated the independent prognostic significance of high TRMT112 expression for poor OS and DSS.

The TME exerts crucial roles in tumor proliferation, invasion, metastasis, angiogenesis, metabolism, immunosuppression, and drug resistance [28, 29]. Being the most important mesenchymal component in the TME, tumor-associated fibroblasts contribute to the pathogenesis of tumors by releasing diverse growth factors, chemokines, and cytokines and participating in the remodeling of the extracellular matrix [30–33]. Here, TRMT112 expression was discovered to exhibit a positive link to tumor-associated fibroblasts in BRCA, ESCA, UCEC, and COAD. Given that increased TRMT112 expression predicted shorter OS and DFS of cancers, we speculated that TRMT112 may participate in the immune response of the abovementioned tumors. Tumor-infiltrating immune cells have been thought to be independently prognostic for tumor metastasis and survival outcome and have been reported to present an intimate relationship with the prognosis of breast cancer [34], lung cancer [35], colorectal cancer [36, 37], and other cancers [38]. Therefore, TRMT112 expression was also analyzed here for its association with several kinds of immune cells in BRCA and LUAD premised on the TIMER database, demonstrating a positive relationship with dendritic cells, CD8+ T cells, neutrophils, CD4+ T cells, B cells, and macrophages. Collectively, it was suggested that high TRMT112 expression acts as a predictor for adverse survival outcomes of cancer patients suffering from LUAD and BRCA and may be regulated by immune infiltration to some extent.

To discuss the molecular mechanism of TRMT112 in tumor occurrence, we combined data on TRMT112-binding protein and TRMT112 expression-associated genes in all cancers. The data revealed that in the majority types of cancers, TRMT112 expression was positively linked to SART1 expression, which was the only common member in the above two groups. As a bicistronic gene, SART1 participates in the initiation and development of HNSC [39] and colorectal cancer [40] and is an essential gene for breast cancer cell division [41]. Hence, it is crucial to further explore whether TRMT112 promotes tumorigenesis and development by interacting with SART1. As oncogenes or tumor suppressor genes [42, 43], noncoding RNAs, such as lncRNA, miRNA, and tRNA, participate in the regulation of cell proliferation [44, 45], apoptosis [46], metastasis [47, 48], differentiation [49], and other biological processes. GO enrichment and KEGG analyses illustrated that TRMT112 might be implicated in tumorigenesis and development through modulating RNA metabolism and transport pathways.

In summary, this is the first study devoted to TRMT112 in pan-cancer, reporting increased expression of TRMT112 in a variety of tumors. TRMT112 may be a potential prognostic predictor. Moreover, our findings also suggest the potential of TRMT112 as an immunomodulatory factor in cancer.

## Data Availability

The gene expression profiles, as well as clinical information, may be accessible on the GDC online platform (https://portal.gdc.cancer.gov/). In this research, publicly accessible datasets were used to conduct the analyses. This information may be accessed at the following links: https://www.oncomine.org, https://xenabrowser.net/datapages/, http://gepia.cancer-pku.cn, https://string-db.org/, http://ualcan.path.uab.edu/analysis-prot.html, http://timer.cistrome.org/, https://cistrome.shinyapps.io/timer/, and https://david.ncifcrf.gov/.

## Supplementary Materials

Supplementary Materials Table S1. Univariate and multivariate COX analyses of TRMT112 expression overall survival (OS) in head and neck squamous cell carcinoma (HNSC). Univariate Cox analysis showed that TRMT112 expression, radiotherapy, initial treatment effect, and lymphatic vascular infiltration were related to OS. Multivariate analysis verified that the elevated TRMT112 expression level independently served as an indicator of the unsatisfactory OS. Table S2. Univariate and multivariate COX analyses of TRMT112 expression for disease-specific survival (DSS) in head and neck squamous cell carcinoma (HNSC). Univariate COX analysis highlighted that TRMT112 expression and initial treatment effect were related to DSS. Multivariate analysis confirmed that high TRMT112 expression was an independent indicator of poor DSS.
