# Supplementary material for: Pan-Cancer Analysis Reveals the Relation between TRMT112 and Tumor Microenvironment
Source: J Oncol. 2022 Aug 30;2022:1445932. doi: 10.1155/2022/1445932 (PMC9448524; doi:10.1155/2022/1445932)
Supplement: Supplementary Materials — Table S1. Univariate and multivariate COX analyses of TRMT112 expression overall survival (OS) in head and neck squamous cell carcinoma (HNSC). Univariate Cox analysis showed that TRMT112 expression, radiotherapy, initial treatment effect, and lymphatic vascular infiltration were related to OS. Multivariate analysis verified that the elevated TRMT112 expression level independently served as an indicator of the unsatisfactory OS. Table S2. Univariate and multivariate COX analyses of TRMT112 expression for disease-specific survival (DSS) in head and neck squamous cell carcinoma (HNSC). Univariate COX analysis highlighted that TRMT112 expression and initial treatment effect were related to DSS. Multivariate analysis confirmed that high TRMT112 expression was an independent indicator of poor DSS. [file 1445932.f1.zip › 1445932.f1/tableS2. HNSC-DSS.docx]

Table S2. Univariate and multivariate COX analysis of TRMT112 expression for disease-specific survival (DSS) in head and neck squamous cell carcinoma

| Characteristics | Total(N) | Univariate analysis | |  | Multivariate analysis | |
| --- | --- | --- | --- | --- | --- | --- |
|  |  | Hazard ratio (95% CI) | P value |  | Hazard ratio (95% CI) | P value |
| TRMT112 (High vs. Low) | 476 | 2.144 (1.496-3.073) | **<0.001** |  | 1.707 (0.997-2.923) | 0.047 |
| Clinical stage (Stage III&Stage IV vs. Stage I&Stage II) | 462 | 1.151 (0.753-1.760) | 0.517 |  |  |  |
| Radiation therapy (Yes vs. No) | 424 | 0.740 (0.492-1.112) | 0.147 |  |  |  |
| Primary therapy outcome (CR vs. PD&SD&PR) | 405 | 0.094 (0.061-0.146) | **<0.001** |  | 0.201 (0.114-0.495) | **<0.001** |
| Gender (Male vs. Female) | 476 | 0.974 (0.656-1.447) | 0.897 |  |  |  |
| Race (White vs. Asian&Black or African American) | 460 | 0.698 (0.412-1.181) | 0.180 |  |  |  |
| Age (>60 vs. <=60) | 476 | 1.078 (0.763-1.524) | 0.670 |  |  |  |
| Smoker (Yes vs. No) | 468 | 1.034 (0.679-1.574) | 0.877 |  |  |  |
| Alcohol history (Yes vs. No) | 466 | 1.212 (0.821-1.789) | 0.334 |  |  |  |
| Lymphovascular invasion (Yes vs. No) | 326 | 1.658 (1.079-2.546) | **0.021** |  | 1.396 (0.857-2.275) | 0.180 |
| Lymphnode neck dissection (Yes vs. No) | 473 | 0.719 (0.465-1.114) | 0.140 |  |  |  |
